# Developing and assessing a density surface model in a Bayesian hierarchical framework with a focus on uncertainty: insights from simulations and an application to fin whales (*Balaenoptera physalus*)

**DOI:** 10.7717/peerj.8226

**Published:** 2020-01-23

**Authors:** Douglas B. Sigourney, Samuel Chavez-Rosales, Paul B. Conn, Lance Garrison, Elizabeth Josephson, Debra Palka

**Affiliations:** 1 Integrated Statistics, Woods Hole, MA, USA; 2 National Marine Mammal Laboratory, NOAA, National Marine Fisheries Service, Alaska Fisheries Science Center, Seattle, United States of America; 3 NOAA Southeast Fisheries Science Center, Miami, FL, United States of America; 4 NOAA Northeast Fisheries Science Center, Woods Hole, MA, United States of America

**Keywords:** Bayesian model, Jagam, Generalized Additive Model (GAM), Tweedie distribution, Fin whales, Density surface model

## Abstract

Density surface models (DSMs) are an important tool in the conservation and management of cetaceans. Most previous applications of DSMs have adopted a two-step approach to model fitting (hereafter referred to as the Two-Stage Method), whereby detection probabilities are first estimated using distance sampling detection functions and subsequently used as an offset when fitting a density-habitat model. Although variance propagation techniques have recently become available for the Two-Stage Method, most previous applications have not propagated detection probability uncertainty into final density estimates. In this paper, we describe an alternative approach for fitting DSMs based on Bayesian hierarchical inference (hereafter referred to as the Bayesian Method), which is a natural framework for simultaneously propagating multiple sources of uncertainty into final estimates. Our framework includes (1) a mark-recapture distance sampling observation model that can accommodate two team line transect data, (2) an informed prior for the probability a group of animals is at the surface and available for detection (i.e. surface availability) (3) a density-habitat model incorporating spatial smoothers and (4) a flexible compound Poisson-gamma model for count data that incorporates overdispersion and zero-inflation. We evaluate our method and compare its performance to the Two-Stage Method with simulations and an application to line transect data of fin whales (*Balaenoptera physalus*) off the east coast of the USA. Simulations showed that both methods had low bias (<1.5%) and confidence interval coverage close to the nominal 95% rate when variance was propagated from the first step. Results from the fin whale analysis showed that density estimates and predicted distribution patterns were largely similar among methods; however, the coefficient of variation of the final abundance estimate more than doubled (0.14 vs 0.31) when detection variance was correctly propagated into final estimates. An analysis of the variance components demonstrated that overall detectability as well as surface availability contributed substantial amounts of variance in the final abundance estimates whereas uncertainty in mean group size contributed a negligible amount. Our method provides a Bayesian alternative to DSMs that incorporates much of the flexibility available in the Two-Stage Method. In addition, these results demonstrate the degree to which uncertainty can be underestimated if certain components of a DSM are assumed fixed.

## Introduction

Anthropogenic use of the world’s oceans is growing at a rapid pace increasing the potential for conflicts with wildlife (Halpern et al., 2008; Bailey et al., 2013) including most species of cetaceans (Stachowitsch, Rose & Parsons, 2019). Density surface models (DSMs) have become valuable tools to help characterize the spatial distribution and abundance of many cetacean species (Forney et al., 2012) and have provided critical information to help guide management decisions in marine environments (Forney et al., 2012; Roberts et al., 2016). The rapid development of techniques for fitting DSMs to data has provided multiple options and the need to evaluate their advantages and limitations.

For cetaceans, DSMs are typically fitted to visual line transect data collected from ships and planes (Redfern et al., 2006; Miller et al., 2013). Fitting DSMs to these data can be challenging because relationships between habitat variables and animal density are often nonlinear and subject to unexplained variance. Another challenge is the observation error associated with the inability to detect all individuals within a surveyed area and therefore the probability of detection needs to be estimated. Because of the diving behavior of cetaceans, not all individuals are available to be seen at the surface at all times. For this reason, the probability of detection of cetaceans involves two components which include (1) the probability of detecting animals at the surface given that they are not diving (i.e., surface detectability) and (2) the probability of animals being at the surface and available for detection (i.e., surface availability). Surface detectability can be estimated using conventional single team distance sampling techniques (Buckland et al., 2001), if the probability of detecting a group on the track line is assumed to be one. If this is not the case, then conventional two-team distance sampling (Laake & Borchers, 2004) can be used or ancillary information is needed to estimate the probability of detecting a group on the track line. Surface availability is not as easily estimated from line transect data alone and usually requires additional information on diving behavior using methods such as those described in Langrock, Borchers & Skaug (2013).

Most previous applications of DSMs have adopted a two-step approach to model fitting (hereafter referred to as the Two-Stage Method), whereby detection probabilities are first estimated using distance sampling detection functions and then subsequently used as an offset when fitting a density-habitat model (Miller et al., 2013). Generalized additive models (GAMs) are commonly used to model spatial distributions due to their flexibility to capture non-linear density-habitat relationships and flexible distributions such as the negative binomial or Tweedie distribution can be adopted to model overdispersion. This method has proven quite robust (Miller et al., 2013; Roberts et al., 2016; Becker et al., 2018) and is currently being used to inform cetacean management in a number of ecosystems (Forney et al., 2012; Roberts et al., 2016; Cañadas et al., 2018; Mannocci et al., 2015).

As an alternative to the Two-Stage Method, hierarchical analysis of distance sampling data has also been developed in the literature (Royle, Dawson & Bates, 2004; Royle & Dorazio, 2008). A number of studies have employed Bayesian techniques to estimate parameters (Eguchi & Gerrodette, 2009; Moore & Barlow, 2011; Conn, Laake & Johnson, 2012). The Bayesian framework is appealing because of its flexibility and ability to take advantage of prior information to inform model output (Clark, 2005). Although examples of applying a Bayesian hierarchical approach to distance sampling are well represented in the literature, model development still lags behind the Two-Stage Method limiting the options available for modelling both the detection function and spatial distribution. For example, applications to line transect data of cetaceans have generally used single team data where detectability on the trackline cannot be estimated directly (Moore & Barlow, 2011; Pardo et al., 2015; Pavanato et al., 2017). There have been fewer attempts to develop a framework that can accommodate two team survey data (but see Conn, Laake & Johnson, 2012). In addition, recent examples of estimating DSMs in a Bayesian framework have used a generalized linear modelling (GLM) framework to parameterize the density-habitat function (Conn, Laake & Johnson, 2012; Pardo et al., 2015; Goyert et al., 2016). This framework is considerably less flexible than the GAM-based models often used in the Two-Stage Method since the latter are based on the use of semi-parametric smoothing functions and better accommodate non-linear species responses to environmental predictors (Guisan, Edwards Jr & Hastie, 2002).

The growth of techniques for predicting the spatial distribution and abundance of animals has led to a number of comparative studies often with a focus on evaluating prediction accuracy among methods (Elith & Graham, 2009; Oppel et al., 2012). Uncertainty estimation, however, is less commonly addressed when evaluating model performance. In fact, in a recent review of studies focused on the distribution and abundance of marine fauna, Robinson et al. (2017) found that uncertainty is rarely assessed or reported. Because models that do not properly account for uncertainty can lead to failure to act or poor management decisions (Taylor et al., 2000) attempts to model distribution and abundance should carefully consider the ability of the chosen method to quantify uncertainty.

In a hierarchical analysis of a DSM, parameters of the density and detection models are estimated jointly such that variance associated with the detection function is propagated throughout the model and represented in the precision of the final estimates (Johnson, Laake & Ver Hoef, 2010). In the Two-Stage Method, variance propagation has historically been somewhat problematic, ranging from no attempt to propagate variance to ad hoc techniques. For example, in some cases a delta method approach has been used (Cañadas et al., 2018) but this relies on a potentially violated assumption of independence among the detection function and density-habitat model (Bravington, Miller & Hedley, 2018). Bootstrap techniques can also be used (Hedley & Buckland, 2004) but lack strong theoretical support (Miller et al., 2013; Bravington, Miller & Hedley, 2018). Williams et al. (2011) introduced an alternative approach where the parameters of the detection function are treated as random effects creating a structure more analogous to the hierarchical approach. Recently, Bravington, Miller & Hedley (2018) modified this method to increase its generality. Although this approach holds promise, there are currently few examples of it being applied in the literature.

Because of the difficulty in propagating uncertainty from the first stage to the second stage when using the Two-Stage Method, it is sometimes not conducted such that final estimates only reflect the uncertainty from the spatial model (Roberts et al., 2016; Palka et al., 2017; Chavez-Rosales et al., 2019). Although it is well understood that this will underestimate uncertainty (Roberts et al., 2016), we are not aware of any studies that have attempted to quantify the degree to which uncertainty is underestimated if variance propagation is ignored. Such information could be of use to managers and other end users of DSMs when trying to incorporate these estimates into the decision making process.

Our study has two main objectives. The first objective is to develop a Bayesian framework, hereafter referred to as the Bayesian Method, for the estimation of DSMs that incorporates the multiple components that influence detection, a flexible distribution that can accommodate overdispersion and a high number of zero observations, and flexibility in the density-habitat function similar to two-stage models. The second objective is to quantify the contribution of each component of a DSM to uncertainty and investigate the degree to which precision is overestimated if the variance of these separate components is omitted. To accomplish these objectives, we use simulations and line transect survey data of fin whales (*Balaenoptera physalus*). We demonstrate the performance of our Bayesian Method in comparison to the Two-Stage Method and compare differences in estimates of precision when variance propagation is not conducted. The results of this study should provide practitioners with an additional tool to conduct DSM analyses and end users with quantifiable information about uncertainty when using the output from currently available DSMs to make management and conservation decisions.

## Materials & Methods

### Data collection

**Figure 1:**
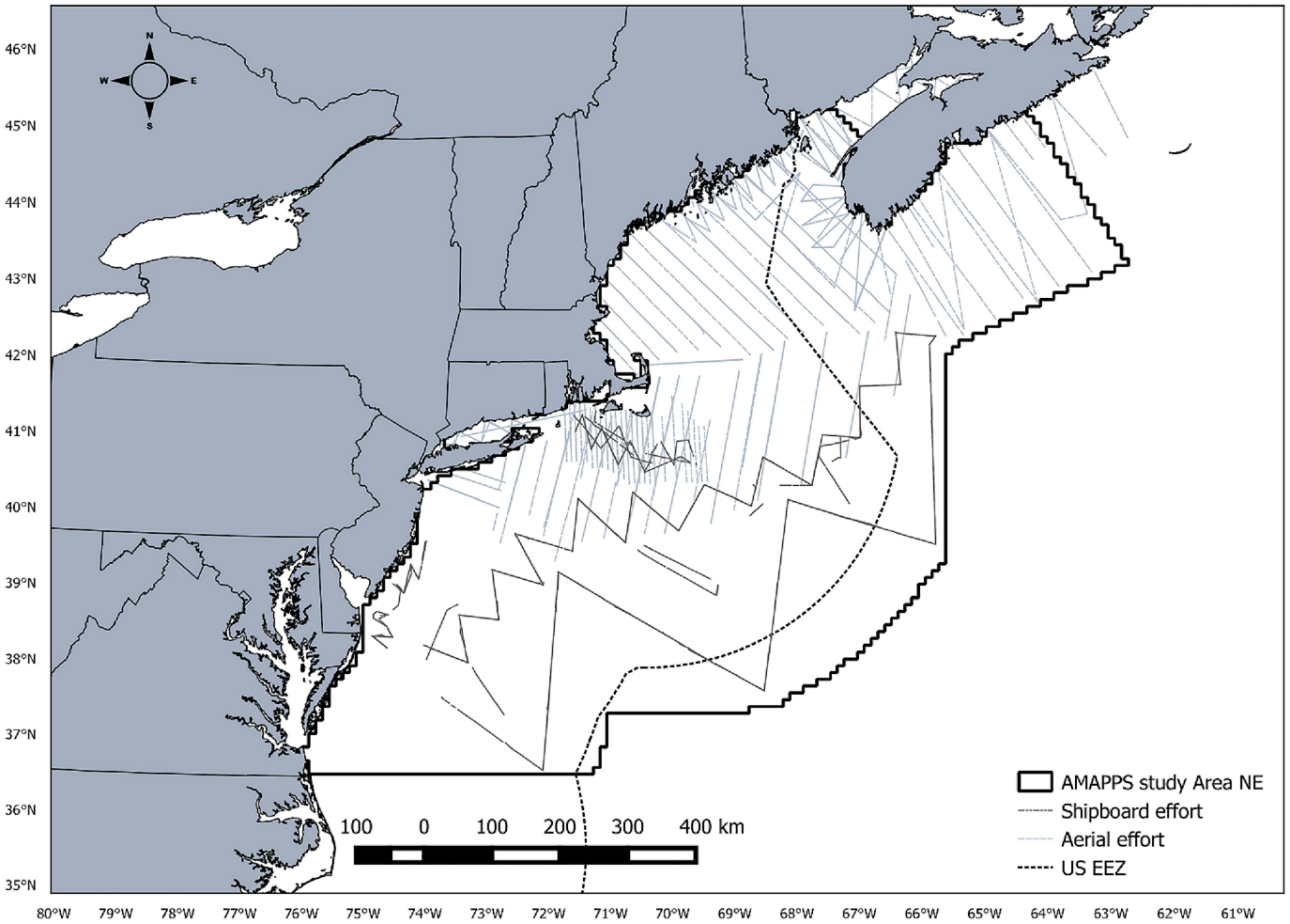
AMAPPS study area. Map of the AMAPPS study area including shipboard survey effort and aerial survey effort for surveys conducted from 2010-2013.

Line transect data were collected as part of the Atlantic Marine Assessment Program for Protected Species (AMAPPS) conducted by the Northeast Fisheries Science Center (NEFSC) and the Southeast Fisheries Science Center (SEFSC). The study area ranges from Halifax, Nova Scotia, Canada to the southern tip of Florida, USA and from the coastline to slightly beyond the US exclusive economic zone covering approximately 1,193,320 km^2^ (Fig. 1). A total of 16 shipboard and aerial surveys combined were conducted from July 2010 to August 2013 covering approximately 104,000 km of line transect survey effort (Table 1). Shipboard surveys were primarily conducted during summer months in offshore waters and aerial surveys were conducted throughout the year primarily in coastal waters. Each survey included two independent observer teams. For the purpose of this study, we only analyzed data collected from the NEFSC surveys and limited the southern extent of the survey area to Cape Hatteras, NC, USA.

**Table 1:** Summary of effort by season and survey.

**Survey**	**Effort (KM)**			
	**Spring**	**Summer**	**Fall**	**Winter**
Shipboard	0	8,146	0	0
Aerial	7,502	10,468	11,038	3,573

We divided the study site into 10 × 10 km grid cells and into 8-day temporal time periods. For each spatial–temporal cell we calculated the amount of on-effort trackline, number of sightings and obtained the corresponding values of a suite of static physiographic variables and dynamic environmental variables (Table S1). Palka et al. (2017) provides more details on the methods to collect and process the line transect and environmental data.

### Model overview

A general form of a DSM for a given unit of a study area can be written as

(1) \begin{eqnarray*}& & E \left( {n}_{i} \right) ={\hat {p}}_{i}{A}_{i}\exp \nolimits ({B}_{0}+\sum _{j}{f}_{j} \left( {X}_{ij} \right) ),\end{eqnarray*}

where $E \left( {n}_{i} \right) $ is the expected number of sightings in unit *i*, ${\hat {p}}_{i}$ represents the probability of detection within the search area of unit *i*, *A*_*i*_ is the amount of area (e.g., km^2^) searched in each unit *i*, $E \left( {n}_{i} \right) $ can represent either the expected number of individuals sighted in unit *i* (Miller et al., 2013) or the expected number of groups sighted in unit *i* (Moore & Barlow, 2011; Pardo et al., 2015), *B*_0_ represents an intercept term and *f*_*j*_ are smooth terms for the environmental covariates *X*_*ij*_.

To fit this model to line transect data using the Two-Stage Method distance sampling (Buckland et al., 2001) is used to estimate ${\hat {p}}_{i}$ which is then multiplied by *A*_*i*_ and the product is used as an offset in a GAM typically using the software *mgcv* (Wood, 2011). Because ${\hat {p}}_{i}$ only includes probability of detection at the surface, it is common practice to further adjust this estimate of detection probability with an estimate of availability at the surface if one exists (Cañadas et al., 2018). If the model is formulated such that the GAM predicts the density of groups then the density of individuals can be calculated by multiplying by an estimate of average group size where group size is simply the number of individuals in a group (Pardo et al., 2015). However, if group size varies spatially a separate spatial model for group size may need to be considered (Redfern et al., 2008).

We take a hierarchical approach to modeling the spatial density of animals where we simultaneously estimate the components of Eq. 1 using MCMC methods (Gelman et al., 2004). We use number of groups sighted in each grid cell as the response variable. To model detection probability we include a detection function model based on distance sampling and an estimate of surface availability. For the density-habitat component we use a Bayesian GAM approach to fitting smooth functions (Wood, 2016). Finally, we also include a submodel for group size to estimate average group size. Below we outline the development of each subcomponent and its implementation in a Bayesian framework. We provide a list of all parameters and definitions in Table S2.

### Detection function

To estimate surface detectability we used information from the double platform survey method. Information collected from this survey design allowed us to apply mark-recapture distance sampling (MRDS) methods (Laake & Borchers, 2004). To model the sightings data from the dual observers we adopt the formulation for point independence outlined by Laake & Borchers (2004). This estimator combines a mark-recapture analysis with conventional distance sampling to estimate detection probability such that detection on the trackline (i.e., *g(0)*) can be estimated directly, and therefore, is not assumed to be 1. The estimator is

(2) \begin{eqnarray*}& & {\hat {\theta }}_{i}=\hat {g} \left( 0,{\mathbi{z}}_{\mathbi{i}} \right) \frac{\int \nolimits \nolimits _{0}^{W}g(y,{\mathbi{z}}_{\mathbi{i}})dy}{W} ,\end{eqnarray*}

where $\hat {g} \left( 0,{\mathbi{z}}_{\mathbi{i}} \right) $ represents the estimate of detection probability on the trackline and is estimated from the double observer data; $g \left( y,{\mathbi{z}}_{\mathbi{i}} \right) $ represents the detection function at distance *y* and is estimated from the distance data; *W* is the truncation distance and **z** is an *g* × *k* matrix of detection covariates that influence surface detectability where *g* is the total number of grid cells and *k* is the total number of detection covariates such as Beaufort sea state, glare, etc. To model $g \left( y,{\mathbi{z}}_{\mathbi{i}} \right) $, we considered half-normal and hazard rate detection functions. To model $g \left( 0,{\mathbi{z}}_{\mathbi{i}} \right) $, we adopted the approach outlined by Laake & Borchers (2004). Specifically, we modeled the binary outcome of whether or not an observer successfully detected an animal group that was present at distance *y* as the outcome of a Bernoulli trial. Further details of analyzing the double platform line transect data are provided in Appendix S1.

For the aerial surveys, the secondary team was positioned toward the back of the plane but had an obstructed view of the trackline complicating a direct implementation of the MRDS approach. Therefore, we estimated an average *g(0)* for the aerial surveys independently where we treated the front team as a single platform and estimated *g(0)* using a trial configuration (i.e., using detections by the rear observers as “trials” for the front observers). The resulting estimate and coefficients of variation (CVs) for *g(0)* was 0.67 (CV = 0.16). We used this estimate to develop an informative prior in the Bayesian Method. Information on estimating *g(0)* and applying it to the aerial data is provided in Appendix S2.

For each survey, we determined the best detection function through a stand-alone MRDS analysis using the *mrds* package in R. A hazard rate likelihood provided the best fit to both the aerial survey data and the shipboard data. Because sample sizes were low for sightings that were positively identified as fin whales, we pooled these sighting with ambiguous sightings that were either a sei whale (*Balaenoptera borealis*) or a fin whale (see Table S3). We compared models using AIC and the top model structure for each survey was used in the Bayesian Method (see Table S3).

### Surface availability

Because most marine animals spend some amount of time below the surface there is a need to also correct for surface availability (*a*) (Laake et al., 1997; Forcada et al., 2004). We developed an informative prior distribution for this parameter using the estimate and corresponding standard error of surface availability for fin whales reported in Palka et al. (2017). Their method for estimating *a* was based on the probability of an animal being at the surface and available for detection during a survey, and took into consideration the species diving and aggregation behaviors, in addition to the amount of time the observer had to analyze any spot of water from each of the survey platforms. This correction tended to be larger for aerial surveys than for shipboard surveys as the window of observation is considerably smaller for aerial surveys. The estimate for fin whales for aerial surveys was 0.37 (CV = 0.34) and assumed to be 1 for shipboard surveys (see Appendix S2 for more details).

Our final survey-specific correction for detection probability in each grid cell *i* is:

(3) \begin{eqnarray*}& & {p}_{i}^{S}={\Theta }_{i}^{S}\end{eqnarray*}

for the shipboard surveys (*S*) and

(4) \begin{eqnarray*}& & {p}_{i}^{A}={\Theta }_{i}^{A}a\end{eqnarray*}

for the aerial surveys (*A*). To investigate possible correlations between the density-habitat model and detection probability model, we also performed a stand-alone analysis of the distance data with the Bayesian Method by omitting the density-habitat model in a manner analogous to the stand-alone MRDS analysis.

### Density-habitat function

We take a GAM approach to parameterize the density-habitat function. The general basis expansion for a single smooth term can be written as

(5) \begin{eqnarray*}& & f \left( x \right) =\sum _{k=1}^{K}{\beta }_{k}{b}_{k}(x),\end{eqnarray*}

where *b*_*k*_(*x*) are basis functions and *β*_*k*_ are parameters to be estimated. The basis size *K* is usually chosen by the user to be large enough to allow an appropriate amount of flexibility in $f \left( x \right) $. To avoid overfitting quadratic penalty terms are included which take the form

\begin{eqnarray*}& & \sum _{k}{\gamma }_{k}{\beta }^{T}{S}_{k}\beta , \end{eqnarray*}

where *S*_*k*_ are penalty matrices and *γ*_*k*_ are smoothing parameters to be estimated.

A multivariate normal distribution can be constructed for the GAM parameters as

\begin{eqnarray*}& & \beta \sim MVN(0,\sum _{k}{\gamma }_{k}{S}_{k}) \end{eqnarray*}

where ∑_*k*_*γ*_*k*_*S*_*k*_ represents a precision matrix (instead of the more common variance–covariance matrix) and the penalty terms are given a vague, gamma prior such as

\begin{eqnarray*}& & {\gamma }_{k}\sim Gamma(0.05,0.005), \end{eqnarray*}

The terms can be estimated efficiently using Gibbs sampling with conjugate priors.

To calculate the precision matrices we used the jagam function in the R package mgcv (Wood, 2016). This function allows the user to specify a number of different smooths (cubic splines, tensor products, etc.) and provides the basic code and input of a JAGS model. In addition, it centers the smooths to facilitate faster convergence.

### Likelihood

We developed a likelihood by assuming count data followed a Tweedie distribution which has been shown to provide a good fit to cetacean data (Miller et al., 2013; Roberts et al., 2016). The Tweedie distribution is a three parameter family of distributions that can take the form of more commonly used distributions such as the normal, Poisson and gamma. If the power parameter (*ρ*) is in the range 1<*ρ*<2 than the distribution can also be referred to as the compound Poisson-gamma (CPG). Because the Tweedie random variables are a sum of *G* gamma variables (*M*) where *G* is Poisson distributed (Jorgensen, 1987), it can be expressed in terms of a Poisson and a gamma distribution such that

\begin{eqnarray*}& & {G}_{i}\sim Poisson({\lambda }_{\mathrm{p}}) \end{eqnarray*}

\begin{eqnarray*}& & {M}_{ij}\sim Gamma(\alpha ,\beta ) \end{eqnarray*}

where

(6) \begin{eqnarray*}& & {\lambda }_{g}= \frac{\alpha }{\beta } \end{eqnarray*}

and

(7) \begin{eqnarray*}& & {Y}_{i}= \left\{ \begin{array}{@{}ll@{}} \displaystyle \sum _{i=1}^{{G}_{i}}{M}_{ij} &\displaystyle {G}_{i}\gt 0\\ \displaystyle 0 &\displaystyle {G}_{i}=0 \end{array} \right. \end{eqnarray*}

where *Y*_*i*_ represents the response variable and the expectation of *Y*_*i*_ is then *E(Y)* = *λ*_p_ *λ*_g_. Lauderdale (2012) shows that under a specific parameterization, the coefficients of the regression model can be estimated by estimating both the Poisson and gamma components separately. Specifically, this parameterization can be written as

(8) \begin{eqnarray*}& & {\lambda }_{P}={e}^{ \frac{\mathbi{X}(\beta -\phi )}{2} }\end{eqnarray*}

(9) \begin{eqnarray*}& & {\lambda }_{g}={e}^{ \frac{\mathbi{X}(\beta +\phi )}{2} }\end{eqnarray*}

where **X** is a design matrix, *β* is a vector of regression coefficients and *ϕ* is a vector of coefficients that control the extent to which the regression coefficients vary between the Poisson component and gamma component of the compound distribution (Lauderdale, 2012).

### Group size

To model group size we use a zero truncated Poisson such that the group size of each sighting is modeled as

\begin{eqnarray*}& & ({s}_{t}-1)\sim Poisson({\lambda }_{s}), \end{eqnarray*}

where *s*_*t*_ is the *t*_*h*_ observation of group size and *λ*_*s*_*+* 1 represents the average group size. This approach assumes that group size is unrelated to detection probability. This assumption is supported by our analysis of fin whale sightings data which did not indicate a strong influence of group size on detection probability. Moore & Barlow (2011) used a similar approach to model group size in their analysis of fin whale data where they also concluded that group size was unrelated to detection probability.

### Density and abundance estimation

The density of individuals within a grid cell is the product of group size, the density of groups in grid cell *i* and the area of grid cell *i* such that

(10) \begin{eqnarray*}& & {N}_{i}= \left( {\lambda }_{s}+1 \right) {D}_{i}{A}_{i}^{G}\end{eqnarray*}

where *D*_*i*_ represents the density of groups in grid cell *i* from the GAM, *λ*_*s*_ + 1 represents the average group size (s), ${A}_{i}^{G}$ is the total area of grid cell *i* and *N*_*i*_ is the predicted number of individuals in grid cell *i*. The total abundance of individuals within the study area is the sum *N*_*i*_ over all grid cells within the study area.

### Model fitting

We fitted the Bayesian Method outlined above using MCMC sampling implemented with the JAGS software (Plummer, 2003). We used vague prior distributions for all parameters with the exception of *g(0)* for the aerial surveys and $\hat {a}$ where we used estimates and associated CVs to develop informative beta prior distributions. We included a burnin of 20,000 samples and two chains of 50,000 with a thinning rate of 50. We assessed convergence by examining trace plots and calculating Gelman–Rubin diagnostics (see Table S4 for results).

### Simulation study

To evaluate the Bayesian Method and compare its performance to the Two-Stage Method we used a simulation study. We simulated spatial variation in abundance among 1,000 hypothetical grid cells. We next simulated line transect sampling in a subset of the grid cells. We fitted both the Bayesian Method and the Two-Stage Method to each of 500 simulated datasets to estimate abundance and compare results to the true abundance used in the simulation. We use these results to quantify bias and precision of each method and evaluate statistical interval coverage. We provide a more detailed summary of the simulation study in Appendix S3.

### Application to fin whales

To further evaluate our modeling approach and investigate sources of uncertainty we analyzed a four year dataset of fin whales sightings collected during the AMAPPS surveys with both the Bayesian Method and the Two-Stage Method. For the Two-Stage Method we used the formulation of Miller et al. (2013) where we used estimates from the stand-alone MRDS analysis as an offset for $\widehat{{p}_{i}}$ and *A*_*i*_ is the same as described above for the Bayesian Method. For the density-habitat model, we fitted GAMs in R using the *mgcv* package version 1.8-28 (Wood, 2014). We used thin plate regression splines and restricted maximum likelihood (REML) to estimate parameters. We set the basis size (*K*) to 5 for all smoothing functions which was large enough to allow enough flexibility in fitting the smooth terms. To account for overdispersion, a Tweedie distribution was assumed. We used a combination of Akaike’s Information Criterion (AIC) and deviance explained to determine the best set of covariates and the best structure of the smooth terms. Because our interest was in keeping as many components of the model structure consistent across methods for the purpose of comparison, we did not perform model selection with the Bayesian Method and instead opted to use the same model formulation for both the detection function and the density-habitat model in the Bayesian Method that we used in the Two-Stage Method.

To compare the Bayesian Method to a scenario where there is no variance propagation, we intentionally did not propagate uncertainty from the first step when applying the Two-Stage Method such that the precision of final estimates only reflected the density-habitat model uncertainty. To further investigate how much each component of a DSM contributes to uncertainty of the final estimate of abundance we ran several parallel analyses with the Bayesian Method where in each analysis we fixed one component from the first step to its point estimate such that the uncertainty in that component was not propagated. For the detection function components, we used point estimates from the stand alone MRDS analysis. Analogous to the Two-Stage Method, we also ran a model in the Bayesian framework where we fixed all components to their point estimates such that only the density-habitat model uncertainty was represented. For each analysis, we calculated an abundance estimate and CV and compared changes in CV to the full Bayesian model.

## Results

### Simulation study

Results from the simulation study showed that the Bayesian Method was able to achieve close to the nominal 95% rate of coverage with low bias. In addition, results were comparable to the Two-Stage Method. Estimated coverage for both methods was 94.9%. The average CV of abundance estimates was 0.16 for the Bayesian Method which was similar to the average CV of 0.14 for the Two-Stage Method although the distribution of CVs were more positively skewed for the Bayesian Method (Fig. S1). Bias was low (<1.5%) for both methods although marginally more negative in the Bayesian Method (Fig. S1).

### Application to fin whales

A comparison of the resulting detection functions between the stand-alone MRDS and the Bayesian Method showed overall detection probabilities were similar although higher in the Bayesian Method (Table 2). For the shipboard surveys, estimates of detectability from the distance sampling component was approximately 13% higher than the estimates from the stand-alone MRDS whereas estimates of *g(0)* were the same. For the aerial surveys estimate of detectability from the distance sampling component was approximately 17% higher than the stand-alone MRDS analysis. Estimates of detection probabilities from the stand-alone Bayesian analysis were closer to the stand-alone MRDS analysis then estimates from the full Bayesian Method for the aerial survey whereas estimates for the shipboard survey were unchanged (Table 2).

**Table 2:** Comparison of posterior estimates for detection functions. Estimates of detection probabilities from the analysis of the fin whales data using the full Bayesian Method with the habitat model included (BM_Full), a stand-alone analysis of the distance data with the Bayesian Method that excludes the habitat model (BM_SA) and a stand-alone analysis of the distance data using the software package mrds in R (MRDS). Results are shown for each survey along with the truncation distance (W) used in eachanalysis. Estimates of detection from the distance sampling component (P_D_) and g(0) from the mark-recapture component are shown with coefficients of variation in parentheses.

Model	Survey	W (km)	P_D_	g(0)
BM_Full	Shipboard	6	0.36 (0.14)	0.66 (0.11)
BM_SA	Shipboard	6	0.35 (0.14)	0.66 (0.11)
MRDS	Shipboard	6	0.30 (0.16)	0.66 (0.13)
BM_Full	Aerial	0.9	0.47 (0.10)	0.67 (0.16)^*^
BM_SA	Aerial	0.9	0.44 (0.14)	0.67 (0.16)^*^
MRDS	Aerial	0.9	0.40 (0.20)	0.67 (0.16)^*^

**Notes.**

* Estimates of g(0) for aerial surveys were taken from Palka et al. (2017).

The posterior estimate of mean group size was 1.4 (CV = 0.14). Most observed group sizes were fewer than 2 animals with approximately 4% greater than 3 animals (Fig. S2).

**Figure 2:**
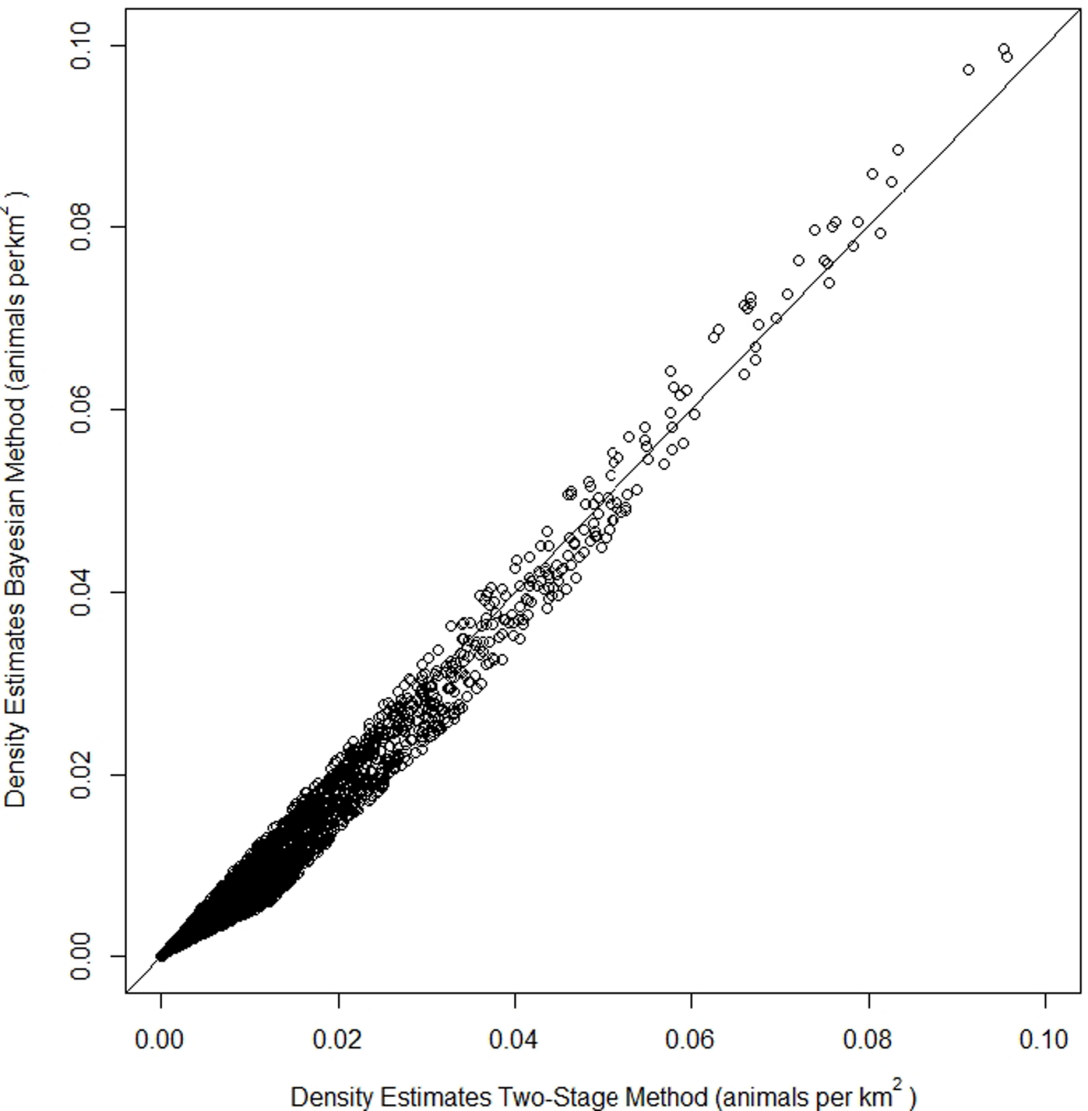
Comparison of density estimates of fin whales (*Balaenoptera physalus*) from a Density Surface Model (DSM) using the Bayesian Method vs density estimates from a DSM using the Two-Stage Method in the Atlantic Marine Assessment Program of Protected Species (AMAPPS) study area in summer.

**Figure 3:**
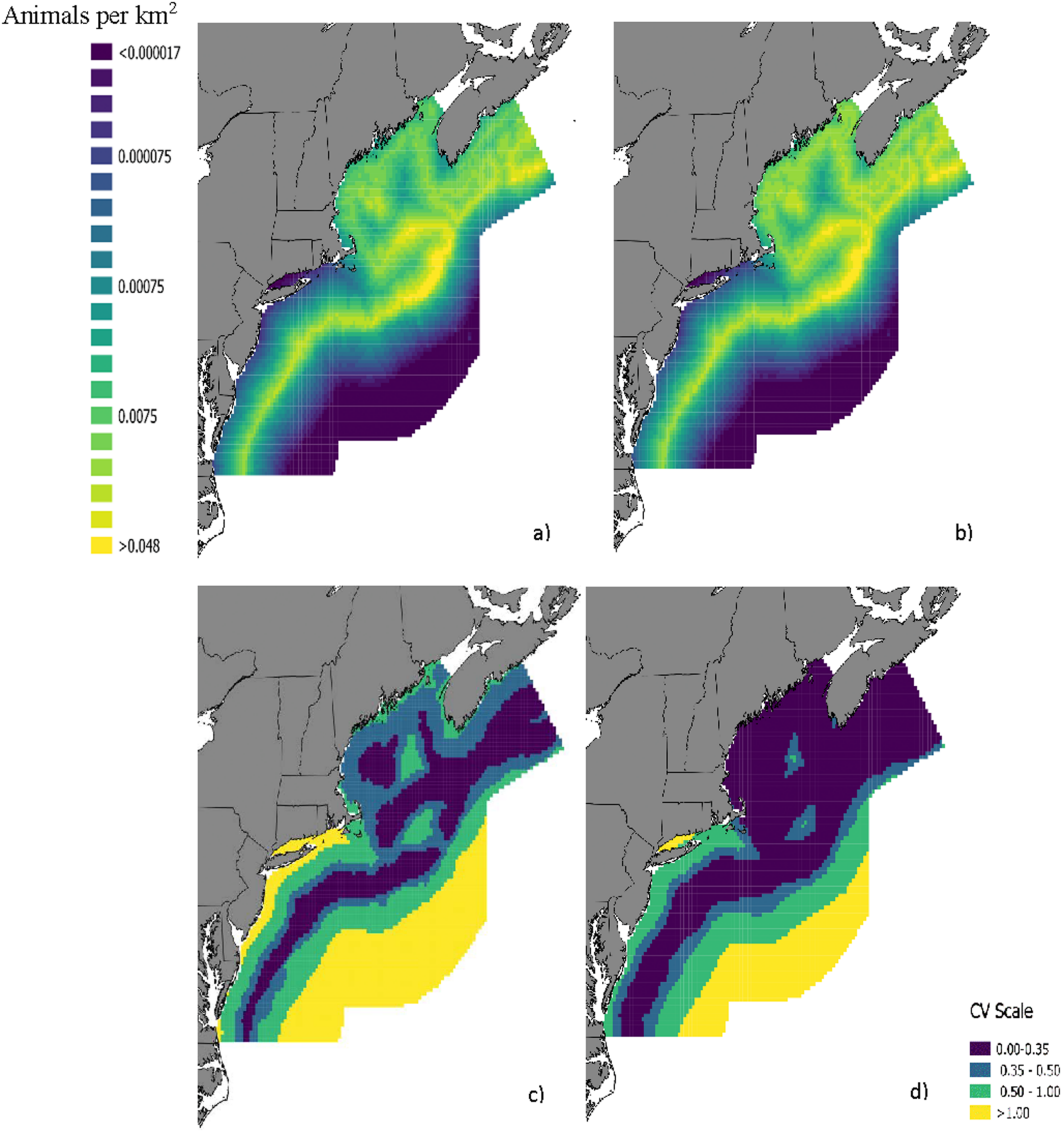
Maps of fin whale density with CVs. Predicted densities of fin whales (*Balaenoptera physalus*) in summer from a density surface model using (A) the Bayesian Method and (B) the Two-Stage Method. Coefficients of variation for the density estimates from (C) the Bayesian Method and d) Two-Stage Method are also provided.

The top model for the density-habitat function included distance to shore, depth, sea surface temperature and distance to 125 m isobath as covariates. Results from fitting the Bayesian Method to the observed sightings data showed good agreement between predicted and observed number of groups per grid cell although there was some tendency of the model to under predict as the number of sightings increased (Fig. S3). In comparison to the Two-Stage Method, density estimates for the grid cells during summer were similar between the two methods (Fig. 2). This similarity in model performance resulted in predicted density distribution patterns that were largely similar among methods (Figs. 3A and 3B). The pattern in uncertainty was also similar for the two methods while the Bayesian Method produced noticeably higher CVs overall (Figs. 3C and 3D).

The analysis of the variance components suggested that variance associated with detectability had a significant influence on the precision of the final abundance estimate whereas variance in mean group size made a negligible contribution (Table 3). The CV for the abundance estimate decreased by 46.9% and 40.6% when we fixed detection probabilities for the aerial survey and shipboard survey to their point estimates, respectively (Table 3). The effect of fixing only surface availability to its point estimates also reduced the CV substantially (Table 3). The CV for the abundance estimate from the full Bayesian Method was greater than 2x the CV from the Two-Stage Method. The abundance estimate and CV for the Bayesian Method were similar to the Two-Stage Method when all components were fixed in the Bayesian Method (Table 3). Abundance estimates of fin whales in the study area ranged from 4012 to 4551 depending on which components were fixed to their point estimates and which method was used (Table 3).

**Table 3:** Maps of fin whale density with CVs. Comparison of abundance estimates ($\hat {\mathbi{N}}$) and corresponding coefficients of variation (${\mathbi{CV }}_{\hat {\mathbi{N}}}$) from Density Surface Models (DSMs) fitted to fin whales (*Balaenoptera physalus*) with specific components of the DSM fixed to their point estimates where ${\hat {\lambda }}_{s}$, $\hat {a}$, ${\hat {p}}^{S}$ and ${\hat {p}}^{A}$ represent the point estimates for average group size, surface availability, detection probability of the ship and detection probability of the plane, respectively. Estimates from the Two-Stage Method and the Bayesian Method with all components fixed (i.e., only the habitat model variance is represented) are also included along with the percent change in ${\mathbi{CV }}_{\hat {\mathbi{N}}}$ when compared to the full Bayesian Method and the prior coefficients of variations of the fixed components (*CV*_*P*_).

Method	Fixed Component	*CV*_*P*_	$\hat {\mathbi{N}}$	${\mathbi{CV }}_{\hat {\mathbi{N}}}$	% change in ${\mathbi{CV }}_{\hat {\mathbi{N}}}$
Bayesian	None	–	4012	0.32	–
Bayesian	${\hat {\lambda }}_{s}$	0.14	4013	0.31	−3.1
Bayesian	$\hat {a}$	0.34	4345	0.21	−34.4
Bayesian	${\hat {p}}^{S}$	0.20	4105	0.19	−40.6
Bayesian	${\hat {p}}^{A}$	0.43	4203	0.17	−46.9
Bayesian	All (except habitat model)	–	4399	0.13	−59.4
Two-Stage	All (except habitat model)	–	4551	0.12	−65.6

## Discussion

We developed and tested a Bayesian formulation of a DSM and compared it with the more commonly used Two-Stage Method. Simulation testing suggested that our model formulation performed well and provided similar results as the Two-Stage Method when applied to field data. Furthermore, we have utilized this hierarchical framework to explore the contribution of different components of detection probability to uncertainty in final abundance estimates. Our analysis demonstrated that changes in precision can be considerable when variance from different detection components is not fully propagated to the final estimates.

Two-step approaches to DSMs have the advantage of being able to use all the built-in options available in different software packages such as Distance (Thomas et al., 2010) to model detection and *mgcv* to model density-habitat relationships (Miller et al., 2013). In contrast*,* Bayesian approaches have been more limited in these options (but see Niemi & Fernández, 2010 for a detailed example). We have taken steps to expand upon previously published Bayesian approaches to DSMs increasing its flexibility. For example, we adopted the MRDS approach available in Distance to model two team data while also including a hazard rate option. Previous Bayesian applications to line transect data of cetaceans have generally been applied to single shipboard team data using a half-normal detection function (Moore & Barlow, 2011; Pavanato et al., 2017). We also included semi-parametric smooths that allow for flexible, data-driven relationships between habitat and density. Other Bayesian implementations of DSMs have included quadratic terms in a GLM framework (Pardo et al., 2015; Goyert et al., 2016) to capture nonlinear relationships, but this approach is still parametric in form and limited in flexibility. Finally, we implemented a Tweedie distribution as a flexible model for count data that allows for both a large number of zeros and overdispersion, two features ubiquitous in animal population surveys. Because the Tweedie is not a built-in distribution in most Bayesian software packages, we adopted the CPG approach of Lecomte et al. (2013). Together these features provide more options for users when fitting Bayesian models to line transect data.

The Bayesian Method performed well in simulation testing and results were comparable to the Two-Stage Method. To compare variance propagation among methods we intentionally restricted our simulations to relatively simple scenarios so we could confidently apply the delta method to the Two-Stage Method without violating assumptions of independence. Bias was low and statistical coverage was close to the nominal 95% rate for both methods suggesting that an appropriate amount of uncertainty was being propagated without being overly conservative. In addition to simulations, the application to fin whale data demonstrated that both methods can achieve similar results in terms of predicted spatial distributions when applied to actual field data. Both models predict high densities around the Gulf of Maine and around the shelf break with lower densities towards the southern boundary of the study area. Furthermore, the resulting abundance estimates and predicted spatial distribution of fin whales off the east coast of the United Sates compare favorably with a recently published two-stage study by Roberts et al. (2016) for the same general area providing further confidence in model performance.

Although overall performance was similar among methods it is important to note that the estimate of fin whale abundance was approximately 11% lower for the Bayesian Method even though we aimed to keep the model structure as similar as possible. This difference may partly be influenced by differences in the detection function. In comparison to the stand-alone MRDS analysis, the Bayesian Method produced higher estimate of detection probability which partly explains why estimates of abundance were lower. Estimates of detection probability parameters from the Bayesian Method also appeared to be correlated with parameters of the density-habitat function, which is certainly possible when detection and density-habitat functions are fitted simultaneously and covariates influencing the two functions are themselves correlated (Bravington, Miller & Hedley, 2018). By contrast, when we fitted a Bayesian model to detection data alone (i.e., did not estimate density), estimates of detectability were much closer to the MRDS analysis (although still somewhat higher). This correlation was most apparent for the aerial surveys which had substantially lower samples size than the shipboard surveys. Because estimating detection probability within the Bayesian Method essentially acts like an informed prior for detection probability, reducing correlation with the density-habitat function will partly be a function of how much data (i.e., sightings) are available to estimate parameters. Although there are methods to prevent feedback in Bayesian graphical models (Plummer, 2015), we did not pursue those options here. Nevertheless, the correlation between detection covariates and habitat covariates is important issue to consider when interpreting the results from a DSM (Bravington, Miller & Hedley, 2018).

Our investigation of variance with the fin whale data demonstrated that changes in the CV of abundance estimates can be substantial if variance is not propagated from specific components of a DSM. In addition to abundance, CV maps of density predictions illustrated considerably higher uncertainty in spatial distribution of fin whales from the Bayesian Method with full variance propagation when compared to the Two-Stage Method which did not include variance propagation. Although this qualitative result is unsurprising, our study establishes the extent to which uncertainty can be underestimated when uncertainty associated with individual components of detection is not propagated into final estimates. Furthermore, by highlighting sources of uncertainty our analysis provides some guidance for which components to target to reduce uncertainty in abundance estimates. For example, recent advances in sampling technology such as passive acoustic technology (Marques et al., 2013) and aerial drones (Brack, Kindel & Oliveira, 2018) may be greatly beneficial in estimating both more accurate and more precise measurements of surface availability and in turn could greatly reduce uncertainty in abundance and density estimates.

There were a number of other factors that could influence uncertainty that we did not investigate. For example, we did not include any model averaging and model selection was only performed using the Two-Stage Method. Model selection could also be performed using the Bayesian Method with a number of different approaches (Hooten & Hobbs, 2015). We chose not to perform Bayesian model selection or model averaging here because our focus was on trying to keep the structure as similar as possible between the two frameworks for the purpose of comparison. In addition to model uncertainty, another factor that could influence uncertainty is spatial autocorrelation. Both methods assumed independence among grid cells and we did not attempt to model the spatial autocorrelation. However, it is possible to directly model the spatial autocorrelation (see Johnson, Laake & Ver Hoef, 2010 and Bachl et al., 2019 for examples). The extent to which these factors can contribute to uncertainty in estimates of density and abundance is an important issue to consider.

Proper consideration of uncertainty is crucial to effective management of natural resources (Ludwig, Hilborn & Walters, 1993). A number of studies have shown how failure to consider uncertainty can result in poor management decisions (Regan et al., 2005; Artelle et al., 2013). For example, in population viability analysis, ignoring error in initial population size may result in misleading estimates of population persistence (Mcloughlin & Messier, 2004). In the management of cetacean populations, overly precise estimates of abundance can have direct consequences on the determination of potential biological removal and may result in a lack of management action when action should be taken (Taylor et al., 2000). Using a Bayesian estimation approach, Gerrodette & Eguchi (2011) demonstrated how a more complete consideration of uncertainty of spatial distribution can result in a more cautionary approach to the design of a marine reserve that may ultimately be more effective for conservation. Taken together, these studies suggest that modeling tools used to inform management decisions must prioritize a full assessment of uncertainty to avoid undesirable outcomes.

## Conclusions

Rigorously quantifying uncertainty in population estimates is a challenging but important goal. Most applications of two-stage DSMs published to date have not propagated uncertainty from all detection components into final abundance estimates, resulting in overly precise estimates. By contrast, incorporation of detection errors in Bayesian approaches such as those implemented here are straightforward. Recently, Bravington, Miller & Hedley (2018) developed alternative methods for propagating multiple sources of detection uncertainty in two-stage line transect DSMs. Their approach appears promising, and we expect it will likely become common practice for those conducting two-stage DSM modeling with line transect data. Nevertheless, one stage hierarchical models may be the only way to resolve certain detection processes—for instance, in cases where species misclassification occurs (e.g., Conn et al., 2013). Thus, we expect to see continued, parallel development of hierarchical models for line transect data together with two-stage DSMs.

##  Supplemental Information

10.7717/peerj.8226/supp-1 Appendix S1 Supplementary information on formulating the detection function using the Bayesian Method

10.7717/peerj.8226/supp-2 Appendix S2 Summary of Estimates of *g(0)* and Surface Availability from Palka et al. (2017)

10.7717/peerj.8226/supp-3 Appendix S3 Additional details on the simulation study

10.7717/peerj.8226/supp-4 Table S1 Environmental Covariates Description of environmental covariates summarized for all grid cells within the Atlantic Marine Assessment Program for Protected Species (AMAPPS) study area. Only distance to the 125 meter isobaths (DIST125), depth (DEPTH), distance to the coastline (DIST2SHORE) and sea surface temperature (SST) were included in the final model to predict densities of fin whales (*Balaenoptera physalus*) within the AMAPPS study area.

10.7717/peerj.8226/supp-5 Table S2 Parameter definitions Equation numbers and definitions for all parameters estimated using the Bayesian Method for both (A) the detection function and (B) habitat function.

10.7717/peerj.8226/supp-6 Table S3 Description of Detection Function Models Description of the final MRDS model used to model surface detectability for each survey (Shipboard or Aerial) used in the analysis of the fin whale (*Balaenoptera physalus*) density surface model. The survey specific distance sampling (DS) model, truncation distance (W), and mark-recapture (MR) model are provided. The total number fin whales sightings (N˙FIWH), ambiguous sighting that were either a sei whale (*Balaenoptera borealis*) or a fin whale (N˙FISE) and the total combined sample sizes used in the analysis (N˙TOTAL) are also provided.

10.7717/peerj.8226/supp-7 Table S4 Posterior Summaries of Parameters Posterior summary of parameters and Gelman-Rubin statistics (G-R) from (A) the detection function and (B) the habitat function from the Bayesian Method. The corresponding survey for each set of detection function parameters is also provided.

10.7717/peerj.8226/supp-8 Figure S1 Simulation Results Figure S1: Summary of simulation results after applying the Bayesian Method and the Two-Stage Method to 500 simulated datasets. Panels a & b represent histograms of coefficients of variation (CV) of abundance estimates from each method and C & D represent bias in estimates of population size.

10.7717/peerj.8226/supp-9 Figure S2 Observed group sizes of fin whales (*Balaenoptera physalus*)

10.7717/peerj.8226/supp-10 Figure S3 Observed vs Expected for the Bayesian Method Plot of number of sightings of groups of fin whales (*Balaenoptera physalus*) per grid (Observed) vs the predicted number (Predicted) from a density surface model using the Bayesian Method. Red line indicates the regression line of Observed vs Predicted.****

10.7717/peerj.8226/supp-11 Supplemental Information 11 Fin whale data used to fit both the Two-Stage Method and Bayesian Method described in Sigourney et al

10.7717/peerj.8226/supp-12 Supplemental Information 12 Environmental covariate values for grid cells Mean covariate values for grid cells within the Atlantic Marine Assessment Program of Protected Species (AMAPPS) study area used to make predictions of fin whale (*Balaenoptera physalus*) densities.

10.7717/peerj.8226/supp-13 Supplemental Information 13 Code to run the Two-Stage Method and the Bayesian Method

10.7717/peerj.8226/supp-14 Supplemental Information 14 Simulation Code

10.7717/peerj.8226/supp-15 Supplemental Information 15 Raw sightings data of fin whales formatted for the Bayesian Method

10.7717/peerj.8226/supp-16 Supplemental Information 16 Raw sightings data for mrds Sightings data to fit the stand-alone detection function models using the R package mrds

## Additional Information and Declarations

### Data Availability

The following information was supplied regarding data availability: Raw data and code to run the Bayesian Method, Two-Stage Method and simulations described in the article are available in the Supplemental Files.
