# Supplementary material for: Developing and assessing a density surface model in a Bayesian hierarchical framework with a focus on uncertainty: insights from simulations and an application to fin whales (Balaenoptera physalus)
Source: PeerJ. 2020 Jan 23;8:e8226. doi: 10.7717/peerj.8226 (PMC6983298; doi:10.7717/peerj.8226)
Supplement: Appendix S1 [file peerj-08-8226-s001.docx]

**Appendix S1: Supplementary information on formulating the detection function using the Bayesian Method**

For our detection function we used information from the double platform survey method. Information collected from this survey design allowed us to apply mark-recapture distance sampling (MRDS) methods to estimate detection probability in each grid cell (Laake & Borchers, 2004). To model the sightings data from the dual observers we adopt the formulation for point independence outlined by Laake & Borchers (2004). This estimator combines a mark-recapture analysis with conventional distance sampling (CDS) to estimate detection probability such that *g(0)* can be estimated directly, and therefore, is not assumed to be 1. The estimator is

$$\hat{P}\left( y,\boldsymbol{Z} \right)=\hat{g}\left( 0, \boldsymbol{Z} \right)*\hat{g}(y,\boldsymbol{Z})$$

Where $\hat{g}\left( 0, \boldsymbol{Z} \right)$ represents the estimate of detection probability on the trackline and is estimated from the mark-recapture data, $\hat{g}\left( y,\boldsymbol{Z} \right)$represents the detection function and is estimated from the distance data, *y* is a vector of observed distances and $\boldsymbol{Z}$ is a matrix of detection covariates that influence perception bias. By assuming that the two observer platforms are independent at *y*=0 this method allows for correlation among observers at distances greater than 0.

*DS Component*

To model the likelihood for the distance data we implemented both half-normal and hazard rate detection functions. The likelihood for the half normal detection function can be written as

$$g\left( y,\boldsymbol{Z} \right)=\exp\left( \frac{{-y}^{2}}{2\sigma^{2}} \right)$$

Where σ represents the scale parameter. The likelihood for the hazard rate detection function is

$$g\left( y,\boldsymbol{Z} \right)=1-\exp\left( \frac{-y}{\alpha} \right)^{-b}$$

where α represents the scale parameter and *b* represent the shape parameter.

The probability density function (pdf) is

$$\hat{f}\left( y,\boldsymbol{Z} \right)=\frac{g(y,\boldsymbol{Z})}{\int_{0}^{W} g(y,\boldsymbol{Z})dy}$$

where *W* represents the right truncation distance. The integral in the equation is referred to as the effective strip width (µ). Under the assumptions of CDS (i.e., *g(0)* =1), when the pdf is evaluated at distance *y*=0

$$\hat{f}\left( 0 \right)=\frac{1}{\mu}$$

We model the variance of the half normal detection function,$\sigma_{D}^{2}$ , as a log-linear combination of detection covariates

$\sigma^{2}=\exp(\theta_{0}+\sum_{c=1}^{C} \theta_{c}*Z_{c})$

where $\theta_{0}$ is an intercept term, $\theta_{c}$ represent the *c* linear coefficients of the detection function and *Z_c_*  represents a design matrix of *c* detection covariates (e.g. beaufort sea state, glare, etc.)

*MR Component*

The DS component described above assumes detection on the trackline equals 1. To estimate g(0) directly we can use the MR data collected from the double observer platform. To do so, we model the binary outcome of whether or not observer *m* successfully detects an animal cluster that was present at distance *y* as the outcome of a Bernoulli trial. To model each p we use a logit link function such that

Logit(p_m_) = α_m_ + α_D_**y* + Σ *α_c_ *Z_c_*

where α_m_ represents the probability of detection on the trackline for observer *m*, α_D_ represents the change in detection probability with distance *y*, and the sum terms represents the linear effect of all other detection covariates (e.g. beaufort sea state, visibility etc.).

Each sighting can be assigned to one of three possible capture histories () that are conditional on a group being sighted. The group can be seen by the primary observer and not by the secondary observer (=1, 0), not seen by the primary observer but seen by the secondary observer (=0,1) or seen by both observers (=1,1). Because the probability of detection is conditional on a group being sighted, we write the conditional likelihood of detection given that an animal is sighted as

$£=\prod_{h=1}^{n} P\left( {}_{h} | detected \right)=\prod_{h=1}^{n} \frac{P({}_{h})}{P_{.}}$

where

$p^{10}=$P(ω = (1,0) | detected) = p_1_ * (1-p_2_)

$p^{01}=$P(ω = (0,1) | detected) = (1-p_1_) * p_2_

$p^{11}=$P(ω = (1,1) | detected) = p_1_ * p_2_

We model each one of these capture histories as the outcome of a Bernoulli trial such that

ω*_h_* ~Bern($p^{h})$

where *h* indicates the capture history (i.e. 10, 01 or 11).

For a given set of detection covariates, the estimator for detectability on the trackline (*y*=0) is computed as

$\hat{g}\left( 0,\boldsymbol{Z} \right)$= p_1_(0, ***Z***)+p_2_(0, ***Z***)- p_1_(0, ***Z***)* p_2_(0, ***Z***)

where

p_m_(0,***Z***)=invlogit(α_m_ + Σ α_c_ * *Z* _c_)

Our final correction for detection probability can be written as:

$\hat{p}_{it}^{.}=\hat{g}\left( 0,\boldsymbol{Z} \right)*\frac{\int_{0}^{w} g\left( y | s.Z \right)dy}{W}$

**REFERENCES**

Laake, J., Borchers, D. in *Advanced distance sampling* (Buckland, S.T., Anderson, D.R., Burnham, K.P., Laake, J.L & Thomas, L. ed.) 108-189 (Oxford University Press 2004).
