# Supplementary material for: Developing and assessing a density surface model in a Bayesian hierarchical framework with a focus on uncertainty: insights from simulations and an application to fin whales (Balaenoptera physalus)
Source: PeerJ. 2020 Jan 23;8:e8226. doi: 10.7717/peerj.8226 (PMC6983298; doi:10.7717/peerj.8226)
Supplement: Appendix S2 [file peerj-08-8226-s002.docx]

**Appendix S2: Summary of Estimates of *g(0)* and Surface Availability from Palka et al. 2017**

**Summary**

To implement the Bayesian Method described in the manuscript we used a number of estimates derived in Palka et al. (2017) to develop informed priors. These estimates include detection probability on the trackline (i.e., *g(0)*) for the aerial surveys and an estimate of surface availability for fin whales (*Balaenoptera physalus*). Below we summarize the methods of Palka et al. (2017) and how informed priors were constructed.

**Estimating *g(0)* for aerial surveys**. Due to the physical limitations within the plane, the front and back teams were not able to search the exact same patch of water precluding the use of double observer mark-recapture distance sample (MRDS) methods that assume all animals are available to both teams. The front team had full coverage where observers were able to search waters from the horizon on the right side of the plane (90°) down to directly under the plane (on the track line; 0°) then over to the horizon on the left side of the plane (90°). The back team had an obstructed view that limited coverage. That is, the back observers searched waters from the horizon on the right side of the plane down under the plane through the track line then over to approximately 30 – 35° from the track line on the left side of the plane. In other words, the back team could not search waters on the left side of the plane from the horizon down to 65 – 70° below the horizon. Since most sightings were detected within 50 – 60° of the track line on either side, the patches of water searched by the front and back teams were only slightly asymmetric. However, to explicitly account for this asymmetry, the perception bias-corrected density for the aerial data was estimated in two steps. The first step was to estimate *g(0)* for the front team using only the data from both teams that were in the area of overlap in a double observer MRDS analysis. The second step was to apply this estimate of *g(0)* for the primary team to the density estimates calculated using only the primary team’s data in a standard single team covariate distance sampling analysis.

**Surface Availability.** Availability bias was accounted for in the density estimate by incorporating an availability correction factor as defined by Laake et al. (1997). The needed species-specific data included: (1) average time at the surface and thus available to be detected; (2) average time below the surface and thus unavailable; and (3) amount of time an animal group remained in view of the observers, which depended on the speed of the observation vessel and the group size of the species of interest. Average surface and dive times for fin whales were estimated using time-at depth data collected from suction cup tags attached to individuals by various investigators (Palka et al., 2017). The amount of time an animal remained in view of the observer was estimated during the NE aerial and shipboard surveys using the times and places groups were initially detected.

**Informed Priors.** To use information on the *g(0)* for the aerial surveys and surface availability in the Bayesian Method the estimates and standard errors derived from Palka et al. (2017) were used to develop informative prior distributions using the same method described in Pardo et al. (2015). Specifically, for a given estimate and standard error a beta prior distribution can be constructed as

*q* ~ Beta(a,b)

a= $\hat{q}$c

b=c(1-$\hat{x}$)

c=$\left[ \frac{\hat{x}(1-\hat{x})}{\left( \hat{x}{CV}_{\hat{x}} \right)^{2}} \right]-1$

where *q* is the quantity of interest, $\hat{q}$ is an estimate of *q* and ${CV}_{\hat{q}}$ is the coefficient of variation of that estimate and a and b are parameters of a Beta distribution.

**REFERENCES**

Laake, J.L., Calambokidis, J., Osmek, S.D. & Rugh, D.J. (1997) Probability of detecting harbor porpoise from aerial surveys: Estimating g(0). *Journal of Wildlife Management*, **61**, 63-75.

Palka, D.L., Chavez-Rosales, S., Josephson, E., Cholewiak, D., Haas, H.L., Garrison, L., Jones, M., Sigourney, D., Waring, G.(retired), Jech, M., Broughton, E., Soldevilla, M., Davis, G., DeAngelis, A., Sasso, C.R., Winton, M.V., Smolowitz, R.J., Fay, G., LaBrecque, E., Leiness, J.B., Dettloff, Warden, M., Murray, K. & Orphanides, C. (2017) Atlantic Marine Assessment Program for Protected Species: 2010- 2014. US Dept. of the Interior, Bureau of Ocean Energy Management, Atlantic OCS Region, Washington, DC. OCS Study BOEM 2017-071. 211 pp

Pardo, M.A., Gerrodette, T., Beier, E., Gendron, D., Forney, K.A., Chivers, S., Barlow, J., Palacios, D.M. 2015 Inferring cetacean population densities from absolute dynamic topography of the ocean in a hierarchical Bayesian framework. *PLoS ONE*, **10**,UNSP e0120727.
