# Supplementary material for: Developing and assessing a density surface model in a Bayesian hierarchical framework with a focus on uncertainty: insights from simulations and an application to fin whales (Balaenoptera physalus)
Source: PeerJ. 2020 Jan 23;8:e8226. doi: 10.7717/peerj.8226 (PMC6983298; doi:10.7717/peerj.8226)
Supplement: Appendix S3 [file peerj-08-8226-s003.docx]

**Appendix S3: Simulation study**

To evaluate our modelling framework and quantify differences in precision and coverage probability between the Two-Stage Method and the Bayesian Method we used a simple simulation study. We simulated variation in abundance over 1000 hypothetical grid cells that were all 100 km^2^ in area. Each grid cell was randomly assigned a habitat covariate value that was simulated from a standard normal distribution. For the linear predictor relating the mean number of whales in each grid cell to the covariate value for that grid cell we chose a quadratic function such that the highest number of animals occurs at intermediate values of the covariate. Given this mean value, the actual number of whales in each grid cell was simulated from a Tweedie distribution with the power parameter set to 1.2. Because this distribution is continuous we discretized the predicted number of whales by rounding to the nearest integer. Within each grid cell we assumed whales were homogeneously distributed.

For each simulation we randomly selected 500 grid cells (i.e., 50 % of the study area). We assumed the number of individuals observed in each grid cell is a function of the amount of survey effort and the detection probability such that

$n_{i}=N_{i}P^{c}\hat{P}$

where $n_{i}$ is the number of individuals observed in grid cell *i*, $N_{i}$ is the true number of individuals in grid cell *i*, $P^{c}$ is the probability of being covered by the survey and is a direct function of the area of the grid cell surveyed and $\hat{P}$ is the probability of being detected given coverage and is the product of both surface detectability and surface availability. We assumed independence among grid cells with no overlap in detections such that whales available for detection in one grid cell are not available for detection in another grid cell. To include variation in the amount of survey effort in each grid cell we randomly varied the amount of trackline effort between 1 and 10 km. The search half-width (W) was set to 5 km so the maximum amount of search effort in a grid cell was 100 km^2^ and *P^c^* varied between 0.1 and 1.

We simulated both surface detectability and surface availability. For surface detectability, we simulated two team observer data using a hazard rate function with separate probabilities of detection on the trackline (i.e., *g(0)*) for each team. Each whale was randomly assigned a distance between 0 and 5 km from the trackline. Group size was set to 1 such that each group consisted of a single animal. For surface availability, we set the true probability of being at the surface to 0.75. We included uncertainty about its true value by simulating an estimate of surface availability from a beta distribution with a coefficient of variation (CV) of 0.05. For the Bayesian Method we used an informed beta prior with the mean set to the simulated estimate of surface availability and the CV set to 0.05.

We applied both the Two-Stage Method and the Bayesian Method to each of 500 independently simulated datasets. To fit the Two-Stage Method we used the R package dsm (Miller at al. 2013). For each simulation, we estimated abundance by summing up the estimated number of animals in each grid cell. We used the delta method to calculate variance for the Two-Stage Method and included the variance in surface availability and g(0) in a manner similar to Cañadas et al. (2018). We estimated coverage probability by determining whether or not the true mean population size fell within the 95% confidence intervals of the Two-Stage Method or the 95% credible region of the posterior estimate of the Bayesian Method. For the Two-Stage Method we calculated lognormal confidence intervals and for the Bayesian Method we calculated 95% credible intervals directly from the posterior distribution using the upper 0.975 and lower 0.025 quantiles. In addition to coverage, we calculated percent relative bias for each simulation and method as ($\hat{N}_{sm}$-$N_{s}$)/$N_{s}$ *100 where $N_{s}$ is the true mean population size for simulation *s* and $\hat{N}_{sm}$ is the estimate of population size for simulation *s* and method *m*.

**REFERENCES**

Cañadas, A., Aguilar de Sotoc, N., Aissie, M., Arcangelif, A., Azzoling, M., B-Nagyh, A., Bearzii, G., Campanaj, I., Chicotek, C., Cottel, C., Crostin, R., Davidt, L., Di Nataleo, A., Fortunaf,C., Frantzisq, A., Garciar, P., Gazok, M., Gutierrez-Xarxas, R., Holcerp, D., Larant, S., Laurianof,G., Lewisv, T. Moulinsw, A., Mussix, B., Notarbartolo di Sciarau, G., Panigadau, S., Pastory, X., Politiu, E., Pulcinif,M., Raga, J. A., Rendell, L., Rossow, M., Tepsich, P., Tomás, J., Tringali, M., and Roger, Th. (2018). The challenge of habitat modelling for threatened low density species using heterogeneous data: The case of Cuvier’s beaked whales in the Mediterranean. *Ecological Indicators*, **85**, 128-136.

Miller, D. L., Burt, L. M., Rexstad, E. A., & Thomas, L. (2013) Spatial models for distance sampling data: recent developments and future directions. *Methods in Ecology and Evolution*, **4**, 1001-1010.
