# Supplementary material for: Developing and assessing a density surface model in a Bayesian hierarchical framework with a focus on uncertainty: insights from simulations and an application to fin whales (Balaenoptera physalus)
Source: PeerJ. 2020 Jan 23;8:e8226. doi: 10.7717/peerj.8226 (PMC6983298; doi:10.7717/peerj.8226)
Supplement: Table S1 — Description of environmental covariates summarized for all grid cells within the Atlantic Marine Assessment Program for Protected Species (AMAPPS) study area. Only distance to the 125 meter isobaths (DIST125), depth (DEPTH), distance to the coastline (DIST2SHORE) and sea surface temperature (SST) were included in the final model to predict densities of fin whales (Balaenoptera physalus) within the AMAPPS study area. [file peerj-08-8226-s004.docx]

**Table S1.** Description of environmental covariates summarized for all grid cells within the Atlantic Marine Assessment Program for Protected Species (AMAPPS) study area. Only distance to the 125 meter isobaths (DIST125), depth (DEPTH), distance to the coastline (DIST2SHORE) and sea surface temperature (SST) were included in the final model to predict densities of fin whales (*Balaenoptera physalus*) within the AMAPPS study area.

| **Abbreviation** | **Resolution** | **Description** | **Source** |
| --- | --- | --- | --- |
| DEPTH | 3 arcsec | Bathymetry (m) | ETOPO1 (Amante and Eakins, 2009) |
| D2S | 0.04º | Distance to coastline (m) | Ocean Color Web |
| SLOPE | 3 arcsec | Seafloor slope (º) | ETOPO1 (Amante and Eakins, 2009) |
| D200 | 1’ | Distance to 200 m isobath | ETOPO1 (Amante and Eakins, 2009) |
| D125 | 1’ | Distance to 125 m isobath | ETOPO1 (Amante and Eakins, 2009) |
| D1000 | 1’ | Distance to 1000 m isobath | ETOPO1 (Amante and Eakins, 2009) |
| DIST2SHORE | 1’ | Distance to the coastline (m) | ETOPO1 (Amante and Eakins, 2009) |
| SST | 0.05º | Sea surface temperature (°C) | SST, GOES Imager, Day and Night, Western Hemisphere (8 Day Composite) NOAA NMFS SWFSC ERD |
| LAT |  | Latitude (º) |  |
| CHL | 0.0125º /0.04166º | Chlorophyll a  (mg m-3) | ERDDAP |
| PP | 0.1º | Primary productivity (mgC m−2 yr−1) | ERDDAP |
| PIC | 4 km | Particulate inorganic carbon (mol m-3) | ERDDAP |
| POC | 4 km | Particulate organic carbon (mg m-3) | ERDDAP |
| BTEMP | 1/12º | Bottom temperature (°C) | HYCOM (Chassignet, *et al.,* 2007) |
