# Supplementary material for: Developing and assessing a density surface model in a Bayesian hierarchical framework with a focus on uncertainty: insights from simulations and an application to fin whales (Balaenoptera physalus)
Source: PeerJ. 2020 Jan 23;8:e8226. doi: 10.7717/peerj.8226 (PMC6983298; doi:10.7717/peerj.8226)
Supplement: Table S2 — Equation numbers and definitions for all parameters estimated using the Bayesian Method for both (A) the detection function and (B) habitat function. [file peerj-08-8226-s005.docx]

**Table S2**: Equation numbers and definitions for all parameters estimated using the Bayesian Method for both a) the detection function and b) habitat function.

| Parameter | Definition |
| --- | --- |
| a)  $\boldsymbol{\sigma}_{\boldsymbol{0}}$ | Intercept term for the scale term from the distance sampling (DS) component of the detection function for the hazard arte detection functions |
| $\boldsymbol{\sigma}_{\boldsymbol{B}}$ | Coefficient for the effect of beaufort sea state on detection probability for scale term from the distance sampling (DS) component of the detection function |
| $\boldsymbol{\sigma}_{\boldsymbol{S}}$ | Coefficient for the effect of subjective weather conditions on detection probability scale from the distance sampling (DS) component of the detection function |
| *b* | Shape parameter for the hazard rate function |
| α_1_ | Marginal detection probability for team 1 from the mark-recapture (MR) component of the detection function |
| α_2_ | Marginal detection probability for team 2 from the mark-recapture (MR) component of the detection function |
| α_D_ | Relationship between distance and detection probability from the mark-recapture (MR) component of the detection function |
| b) |  |
| β_0_ | Intercept term for the linear predictor of the habitat function |
| β_D125_1_ | First coefficient for distance to the 125 meter isobath (D125) from the linear predictor used to parameterize the habit function |
| β_D125_2_ | Second coefficient for distance to the 125 meter isobath (D125) from the linear predictor used to parameterize the habit function |
| β_D125_3_ | Third coefficient for distance to the 125 meter isobath (D125) from the linear predictor used to parameterize the habit function |
| β_D125_4_ | Fourth coefficient for distance to the 125 meter isobath (D125) from the linear predictor used to parameterize the habit function |
| Β_DEPTH1_ | First coefficient for depth (DEPTH) from the linear predictor used to parameterize the habit function |
| Β_DEPTH2_ | Second coefficient for depth (DEPTH) from the linear predictor used to parameterize the habit function |
| Β_DEPTH3_ | Third coefficient for depth (DEPTH) from the linear predictor used to parameterize the habit function |
| Β_DEPTH4_ | Fourth coefficient for depth (DEPTH) from the linear predictor used to parameterize the habit function |
| Β_DIST2SHORE1_ | First coefficient for distance to the coast line (DIST2SHORE) from the linear predictor used to parameterize the habit function |
| β_DIST2SHORE2_ | Second coefficient for distance to the coast line (DIST2SHORE) from the linear predictor used to parameterize the habit function |
| β_DIST2SHORE3_ | Third coefficient for distance to the coast line (DIST2SHORE) from the linear predictor used to parameterize the habit function |
| β_DIST2SHORE4_ | Fourth coefficient for distance to the coast line (DIST2SHORE) from the linear predictor used to parameterize the habit function |
| β_SST1_ | First coefficient for sea surface temperature (SST) from the linear predictor used to parameterize the habit function |
| β_SST2_ | Second coefficient for sea surface temperature (SST) from the linear predictor used to parameterize the habit function |
| β_SST3_ | Third coefficient for sea surface temperature (SST) from the linear predictor used to parameterize the habit function |
| β_SST4_ | Fourth coefficient for sea surface temperature (SST) from the linear predictor used to parameterize the habit function |
