# Supplementary material for: Developing and assessing a density surface model in a Bayesian hierarchical framework with a focus on uncertainty: insights from simulations and an application to fin whales (Balaenoptera physalus)
Source: PeerJ. 2020 Jan 23;8:e8226. doi: 10.7717/peerj.8226 (PMC6983298; doi:10.7717/peerj.8226)
Supplement: Table S3 — Description of the final MRDS model used to model surface detectability for each survey (Shipboard or Aerial) used in the analysis of the fin whale (Balaenoptera physalus) density surface model. The survey specific distance sampling (DS) model, truncation distance (W), and mark-recapture (MR) model are provided. The total number fin whales sightings (N˙FIWH), ambiguous sighting that were either a sei whale (Balaenoptera borealis) or a fin whale (N˙FISE) and the total combined sample sizes used in the analysis (N˙TOTAL) are also provided. [file peerj-08-8226-s006.docx]

**Table S3**: Description of the final MRDS model used to model surface detectability for each survey (Shipboard or Aerial) used in the analysis of the fin whale (*Balaenoptera physalus*) density surface model. The survey specific distance sampling (DS) model, truncation distance (W), and mark-recapture (MR) model are provided. The total number fin whales sightings (N_FIWH_), ambiguous sighting that were either a sei whale (*Balaenoptera borealis*) or a fin whale (N_FISE_) and the total combined sample sizes used in the analysis (N_TOTAL_) are also provided.

| Survey | DS Model | W (m) | MR Model | N_FIWH_ | N_FISE_ | N_TOTAL_ |
| --- | --- | --- | --- | --- | --- | --- |
| Shipboard | Distance +Beaufort + Subjective | 6000 | Distance | 111 | 33 | 144 |
| Aerial | Distance +Beaufort | 900 | None | 30 | 6 | 36 |
