# Supplementary material for: Developing and assessing a density surface model in a Bayesian hierarchical framework with a focus on uncertainty: insights from simulations and an application to fin whales (Balaenoptera physalus)
Source: PeerJ. 2020 Jan 23;8:e8226. doi: 10.7717/peerj.8226 (PMC6983298; doi:10.7717/peerj.8226)
Supplement: Table S4 — Posterior summary of parameters and Gelman-Rubin statistics (G-R) from (A) the detection function and (B) the habitat function from the Bayesian Method. The corresponding survey for each set of detection function parameters is also provided. [file peerj-08-8226-s007.docx]

Table S4: Posterior summary of parameters and Gelman-Rubin statistics (G-R) from a) the detection function and b) the habitat function from the Bayesian Method. The corresponding survey for each set of detection function parameters is also provided.

| Parameter | Survey | Median | Mean | SD | low | high | G-R |
| --- | --- | --- | --- | --- | --- | --- | --- |
| a)  $\boldsymbol{\sigma}_{\boldsymbol{0}}$ | Shipboard | -3.37 | -3.35 | 1.32 | -5.88 | -0.77 | <1.01 |
| $\boldsymbol{\sigma}_{\boldsymbol{B}}$ | Shipboard | 0.07 | 0.06 | 0.17 | -0.29 | 0.39 | <1.01 |
| $\boldsymbol{\sigma}_{\boldsymbol{S}}$ | Shipboard | 1.24 | 1.24 | 0.35 | 0.57 | 1.92 | <1.01 |
| *b* | Shipboard | 1.58 | 1.60 | 0.29 | 1.09 | 2.26 | <1.01 |
| α_1_ | Shipboard | -0.34 | -0.34 | 0.25 | -0.85 | 0.15 | <1.01 |
| α_2_ | Shipboard | -0.34 | -0.34 | 0.25 | -0.85 | 0.15 | <1.01 |
| α_D_ | Shipboard | -0.13 | -0.13 | 0.13 | -0.39 | 0.12 | <1.01 |
| $\boldsymbol{\sigma}_{\boldsymbol{0}}$ | Aerial | 0.31 | 0.42 | 1.12 | -1.86 | 2.88 | <1.01 |
| $\boldsymbol{\sigma}_{\boldsymbol{B}}$ | Aerial | -0.56 | -0.64 | 0.39 | -1.6 | -0.05 | <1.01 |
| *b* | Aerial | 1.83 | 1.98 | 0.86 | 0.78 | 4.00 | <1.01 |
| b)  β_0_ | - | -6.39 | -6.40 | 0.39 | -7.19 | -5.67 | 1.02 |
| β_D125_1_ | - | 0.01 | 0.02 | 0.26 | -0.45 | 0.60 | <1.01 |
| β_D125_2_ | - | 0.02 | 0.06 | 0.65 | -1.29 | 1.60 | <1.01 |
| β_D125_3_ | - | -0.07 | -0.13 | 1.68 | -3.74 | 3.30 | <1.01 |
| β_D125_4_ | - | -1.90 | -1.92 | 0.64 | -3.24 | -0.79 | <1.01 |
| Β_DEPTH1_ | - | 0.00 | 0.01 | 0.19 | -0.33 | 0.38 | <1.01 |
| Β_DEPTH2_ | - | -0.01 | 0.05 | 0.60 | -1.04 | 1.37 | <1.01 |
| Β_DEPTH3_ | - | -0.04 | -0.09 | 0.76 | -1.88 | 1.29 | <1.01 |
| Β_DEPTH4_ | - | 0.95 | 0.94 | 0.39 | 0.22 | 1.67 | <1.01 |
| Β_DIST2SHORE1_ | - | -0.01 | -0.01 | 0.28 | -0.55 | 0.65 | <1.01 |
| β_DIST2SHORE2_ | - | 0.04 | 0.17 | 0.63 | -0.68 | 2.05 | <1.01 |
| β_DIST2SHORE3_ | - | 0.29 | 0.74 | 1.67 | -1.13 | 5.40 | <1.01 |
| β_DIST2SHORE4_ | - | 0.93 | 0.88 | 0.50 | -0.50 | 1.7 | <1.01 |
| β_SST1_ | - | 0.00 | 0.02 | 0.12 | -0.14 | 0.28 | 1.02 |
| β_SST2_ | - | 0.00 | -0.01 | 0.22 | -0.36 | 0.32 | 1.02 |
| β_SST3_ | - | 0.00 | -0.01 | 0.32 | -0.56 | 0.56 | 1.02 |
| β_SST4_ | - | -0.17 | -0.19 | 0.20 | -0.62 | 0.12 | 1.02 |
