# Supplementary figures and images for: Developing and assessing a density surface model in a Bayesian hierarchical framework with a focus on uncertainty: insights from simulations and an application to fin whales (Balaenoptera physalus)
Source: PeerJ. 2020 Jan 23;8:e8226. doi: 10.7717/peerj.8226 (PMC6983298; doi:10.7717/peerj.8226)

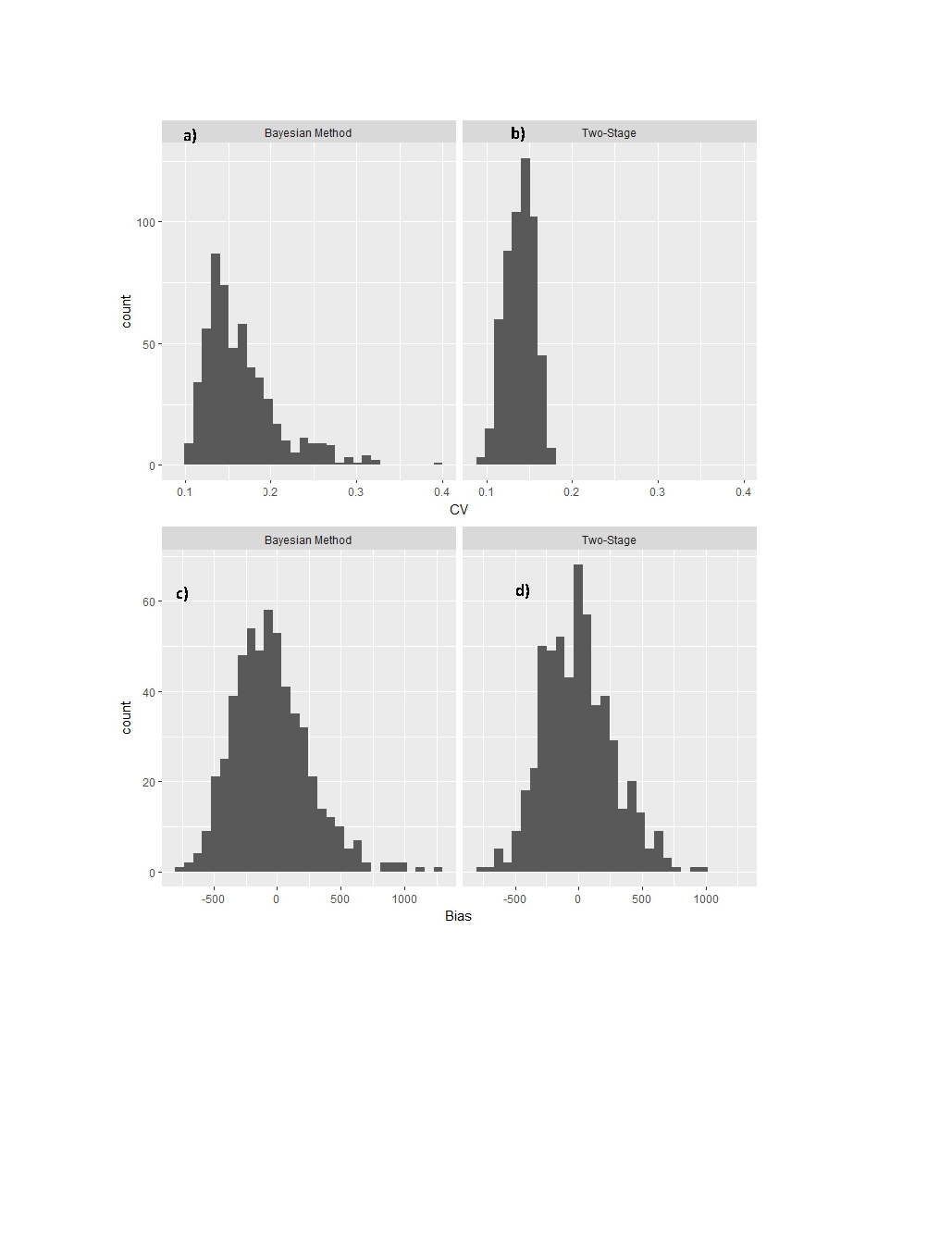

Supplement: Figure S1 — Summary of simulation results after applying the Bayesian Method and the Two-Stage Method to 500 simulated datasets. Panels a & b represent histograms of coefficients of variation (CV) of abundance estimates from each method and C & D represent bias in estimates of population size. [file peerj-08-8226-s008.png]

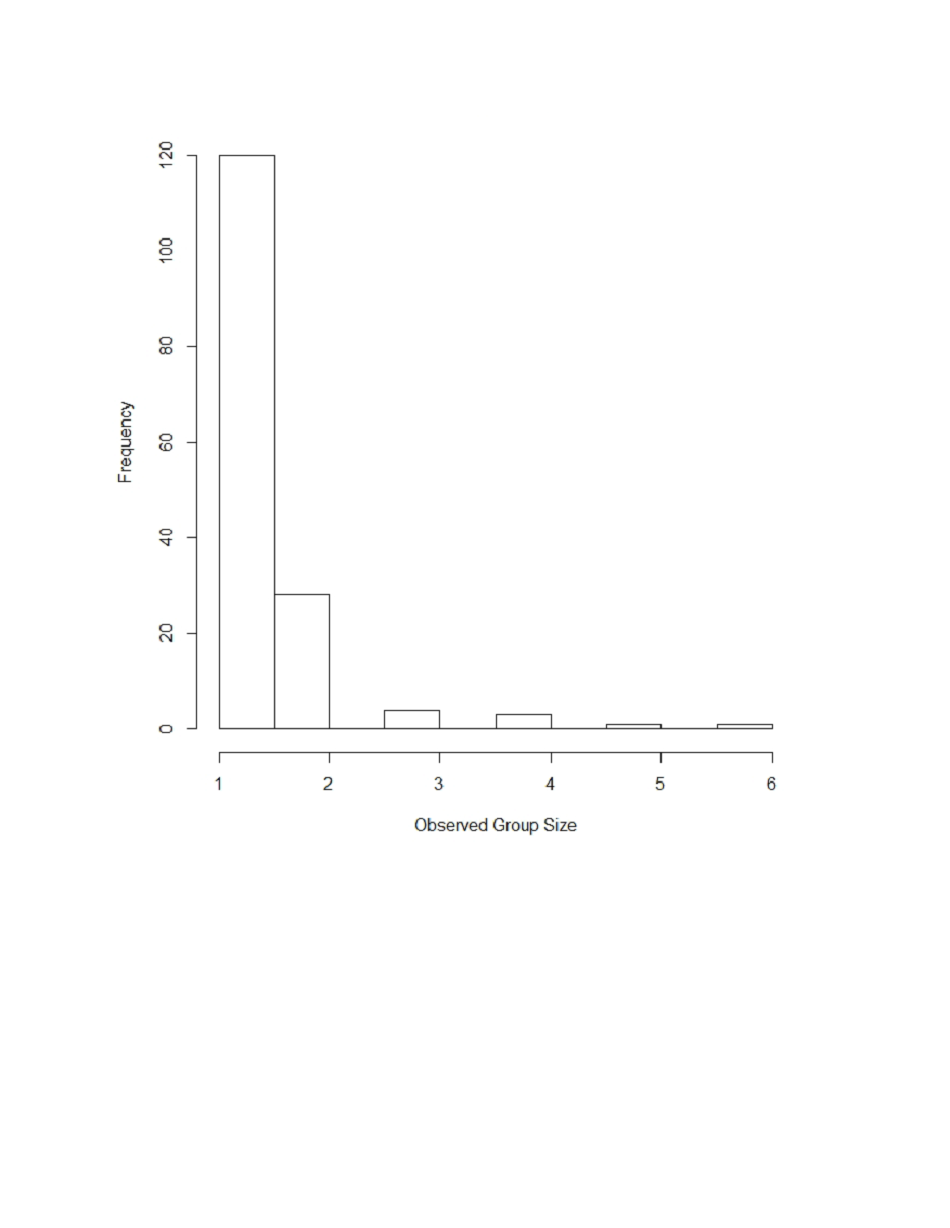

Supplement: Figure S2 [file peerj-08-8226-s009.png]

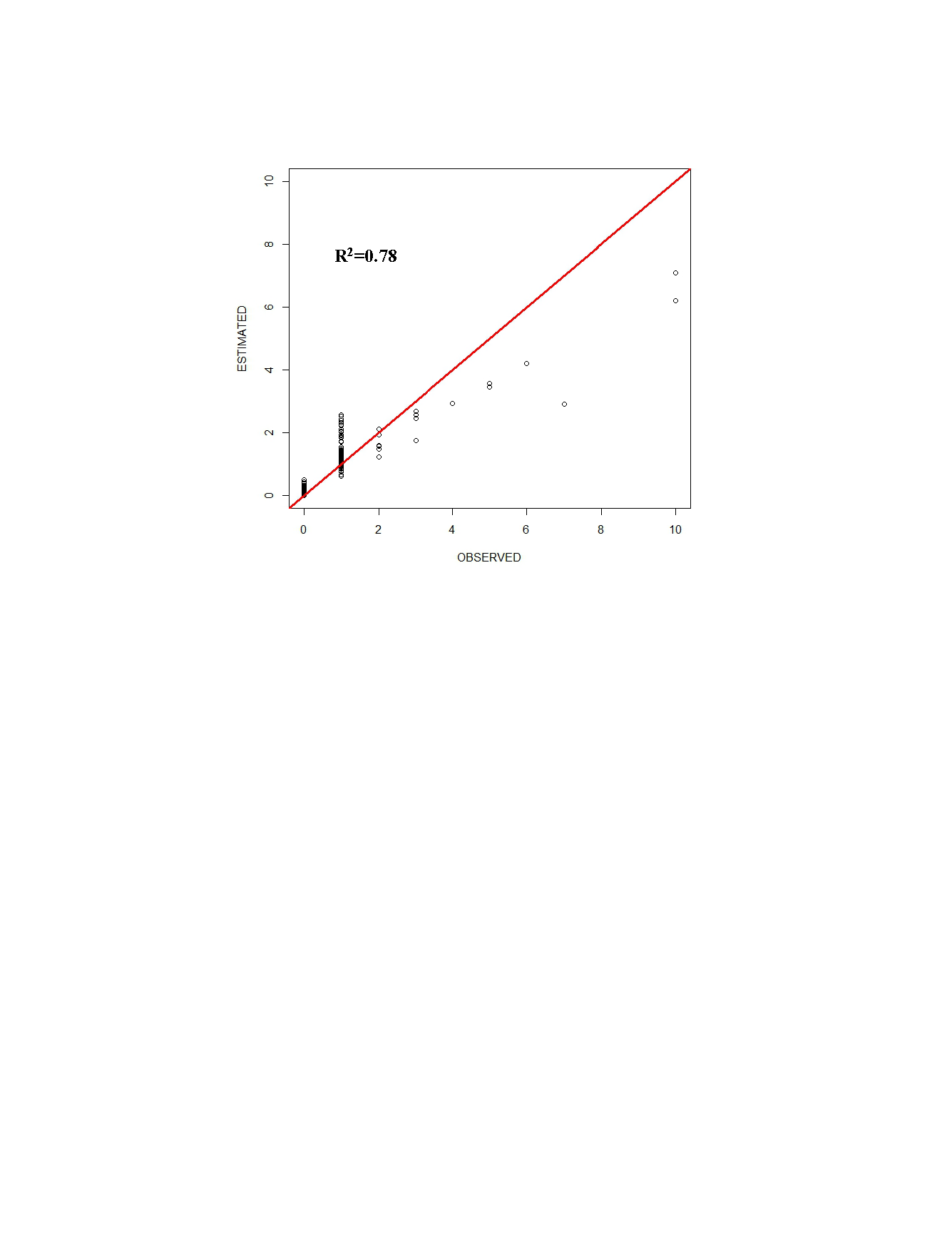

Supplement: Figure S3 — Plot of number of sightings of groups of fin whales (Balaenoptera physalus) per grid (Observed) vs the predicted number (Predicted) from a density surface model using the Bayesian Method. Red line indicates the regression line of Observed vs Predicted. [file peerj-08-8226-s010.png]
